# Commentary: The amygdaloid body of the family Delphinidae: a morphological study of its central nucleus through calbindin-D28k

**DOI:** 10.3389/fnana.2026.1718530

**Published:** 2026-01-23

**Authors:** Luã Carlos de Souza, Paulo Leonardo Araújo de Góis Morais, Adhil Bhagwandin, José Rodolfo Lopes de Paiva Cavalcanti

**Affiliations:** 1 Laboratory of Experimental Neurology, Department of Biomedical Sciences, Faculty of Health Sciences, State University of Rio Grande do Norte (UERN), Mossoró, Rio Grande do Norte, Brazil; 2 Division of Clinical Anatomy and Biological Anthropology, Department of Human Biology, Faculty of Health Sciences, University of Cape Town, Cape Town, South Africa

**Keywords:** amygdala, amygdaloid complex, calbindin-D28k, comparative neuroanatomy, dolphin

The amygdala is a structure located in the medial temporal lobe of humans, dolphins and other mammals (Sacchini et al., 2022) and in the telencephalic homologous nuclei in other vertebrates such birds (Cheng et al., 1999; Tian et al., 2022). Its name originates from Greek and refers to its almond-shaped structure. It was first identified as a distinct brain structure in the early 19th century by Burdach (1819-1822), Klüver and Bucy (1938), Weiskrantz (1956), LeDoux (2000), and LeDoux (2007). It is a critical structure for the integration of sensory and affective information originating from cortical and subcortical regions. Functionally, it plays a crucial role in mediating fear-related behaviors and other mechanisms associated with learning, attention, decision-making and pain perception (LeDoux, 2000; Ochsner et al., 2002; Hsu et al., 2005; Fitzgerald et al., 2006; Adolphs, 2010; Bzdok et al., 2012; Ousdal et al., 2014; Bombardi et al., 2025).

Clinically, in human and animal models, amygdaloid dysfunction is associated with various neurological diseases and psychiatric disorders (Sims and Williams, 1990; Pitkänen and Amaral, 1991), including anxiety and depression (Leppänen, 2006), panic disorder (Wiest et al., 2006; Nardi et al., 2009), and stress-related conditions (Fowler et al., 2017). Regarding dolphins, although the literature reports no clinical evidence of amygdaloid dysfunction, as noted by Sacchini et al. (2022), the unusually enlarged amygdala may instead represent functional adaptations associated with the processing of complex auditory information, in line with the highly specialized acoustic abilities of these animals.

In primates, the amygdala—also referred to as the amygdaloid complex—is typically subdivided into cortical regions and 13 interconnected nuclei. These nuclei, in turn, exhibit further subdivisions characterized by complex intranuclear and internuclear connections. This organization is defined based on cytoarchitectonic, histochemical, and neural connectivity criteria (Krettek and Price, 1978; Pitkänen and Amaral, 1991; de Góis Morais et al., 2021). Cytoarchitectonic descriptions of the amygdaloid complex mostly have emerged from studies in laboratory rodents, primates and humans, with preferential nomenclature usage from laboratory rodents. According to this classification, the amygdaloid complex consists of the amygdaloid body and the extended amygdala. The amygdaloid body is comprised of the basolateral group, the corticomedial or superficial group, the centromedial group, and other amygdaloid nuclei (this includes the intercalated cell masses/islands and the amygdalohippocampal nucleus) (Price et al., 1987; McDonald, 1998; Imam et al., 2022). The dolphinidae amygdaloid complex, like other mammals, is comprised of 12 subnuclei forming three major groups namely, the basolateral complex (or deep nuclei), the cortical or superficial areas and the remaining areas (see details in Sacchini et al., 2024).

The central nucleus of the amygdaloid complex is functionally important since it has previously been assigned a critical role in generating physiological and behavioral responses associated with negative emotions, such as fear and anxiety (Davis, 1997). This nucleus is rich in connectivity, particularly its extensive projections to autonomic and endocrine centers located in the brainstem and hypothalamus (Sorvari et al., 1996).

In this context, the study by Sacchini et al. (2024) employed Nissl staining and calbindin-D28k immunohistochemistry to precisely investigate the neuroanatomy of the amygdaloid complex, with a specific focus on the central nucleus, in the brains of five dolphins from three different species belonging to the Delphinidae family.

The study by Sacchini et al. represents the first classification of the amygdaloid complex in striped, common, and pantropical spotted dolphin species. All three Delphinidae species exhibited amygdaloid complexes that were neuroanatomically similar to those previously described. It is important to note that the central nucleus appears to have various subdivisions across some mammalian species. In the long-tailed macaque (*Macaca fascicularis*) and humans, the central nucleus has two subdivisions (Pitkänen and Amaral, 1993; Pitkänen and Kemppainen, 2002), while three subdivisions have been observed in the common marmoset (*Callithrix jacchus)* (Morais et al., 2019), and up to four have been described in the wistar rat (*Rattus norvegicus*) (Kemppainen and Pitkänen, 2000). Sacchini et al. (2024) identified two distinct subdivisions of the central nucleus in two out of three dolphin species: one located lateral and dorsal to the lateral nucleus, and the other medial and dorsal to the magnocellular division of the basal nucleus, although the medial subdivision was absent in the Atlantic spotted dolphin.

Using cytoarchitectonic techniques, the authors observed morphologically distinct neurons (large, polygonal, round, and fusiform) that were distributed unevenly throughout the central nucleus. The authors then utilized calbindin-D28K immunohistochemistry to identify and define the boundaries of the central nucleus, as previously described by Graïc et al. (2024).

The relevance of the work conducted by Sacchini et al. lies in the fact that a precise categorization of the amygdala, particularly its central nucleus, is indispensable for a clearer and more accurate understanding of one of the most complex functions expressed by vertebrates: emotions. Consequently, the description of the central nucleus morphology, boundaries, and cytoarchitecture may broaden the understanding of how the amygdala modulates emotional experience through its extensive connections with other brain areas.

Studies from human patients with generalized anxiety disorder appear to exhibit extensive amygdala dysfunction within a wide range of involved networks, which can lead to abnormal emotional and cognitive processing (Du et al., 2021). Furthermore, studies point to structural and functional abnormalities in the amygdala associated with suicidal behavior in patients with major depressive disorder (Wang et al., 2020). It is clear form clinical presentations that amygdaloid dysfunction leads to pronounced behavioral changes. It is unclear how changes in amygdaloid anatomy may affect amygdaloid function. Perhaps one way to approach this is to broaden our comparative understanding of the amygdaloid complex and potentially infer changes in anatomical presentation and associated behavior to those observed in clinical presentations.

It is important to note that various neurotransmitters, such as serotonin (5-hydroxytryptamine) and ɤ-aminobutyric acid (GABA) have previously been associated with these clinical conditions, described above. For example, medications that act by blocking the reuptake of serotonin —a first-line treatment for depression and anxiety— has been shown to negatively influence amygdala activation in response to emotional stimuli (Bigos et al., 2008; Murphy et al., 2009; Godlewska et al., 2012). Additionally, reduced GABA-eric inhibition of the basolateral amygdaloid nucleus has been associated with behavioral hyperexcitability, thus potentially increasing anxiety and depression levels, emotional dysregulation, and the development of seizure activity (Prager et al., 2016). In this regard, Salamanca et al. (2024) demonstrated in rats the co-existence of VIP- and GABA-ergic inhibitory neurons, suggesting that VIP-immunoreactive cells may modulate excitatory pyramidal neurons as well as other inhibitory populations in a region-specific manner within the amygdaloid complex, providing an additional layer of inhibitory control relevant to emotional and cognitive processing.

Therefore, considering the structural similarities between the amygdaloid complex of dolphins and primates, it is possible that both orders may share convergent amygdaloid neuroanatomical organization potentially related to emotional information processing, specifically regarding responses associated with fear and anxiety. To further explore whether such structural convergence is accompanied by comparable connectivity patterns, studies employing diffusion tensor imaging, such as the work of Graïc et al. (2023) in sheep, demonstrate that this approach can effectively map amygdaloid networks even in non-conventional species, highlighting its potential value for future investigations in cetaceans. However, this remains to be thoroughly explored in dolphins.

Finally, we duly respect the neuroanatomical diversity of the amygdaloid complex and acknowledge the challenges in accepting consensus regarding the anatomical boundaries of the various amygdaloid nuclei. Additionally, we recognize that there are varied methodological approaches in characterizing neuronal types. Thus, we temper our conclusions and suggestions accordingly.
